# The microbial community characteristics of ancient painted sculptures in Maijishan Grottoes, China

**DOI:** 10.1371/journal.pone.0179718

**Published:** 2017-07-05

**Authors:** Yulong Duan, Fasi Wu, Wanfu Wang, Dongpeng He, Ji-Dong Gu, Huyuan Feng, Tuo Chen, Guangxiu Liu, Lizhe An

**Affiliations:** 1 Key Laboratory of Extreme Environmental Microbial Resources and Engineering, Gansu Province, Northwest Institute of Eco-Environment and Resources, University of Chinese Academy of Sciences, Lanzhou, P.R.China; 2 National Research Center for Conservation of Ancient Wall Paintings and Earthen Sites, Dunhuang Academy, Dunhuang, Gansu, P.R.China; 3 MOE Key Laboratory of Cell Activities and Stress Adaptations, School of Life Sciences, Lanzhou University, Lanzhou, P.R.China; 4 Laboratory of Environmental Microbiology and Toxicology, School of Biological Sciences, The University of Hong Kong, Hong Kong SAR, P.R.China; Wadsworth Center, UNITED STATES

## Abstract

In this study, a culture-independent Illumina MiSeq sequencing strategy was applied to investigate the microbial communities colonizing the ancient painted sculptures of the Maijishan Grottoes, a famous World Cultural Heritage site listed by UNESCO in China. Four mixed samples were collected from Cave 4–4 of the Maijishan Grottoes, the so-called Upper Seven Buddha Pavilion, which was built during the Northern Zhou Dynasty (557-581AD). The 16/18S rRNA gene-based sequences revealed a rich bacterial diversity and a relatively low fungal abundance, including the bacterial groups Actinobacteria, Acidobacteria, Bacteroidetes, Cyanobacteria, Chloroflexi, Firmicutes, Proteobacteria and Verrucomicrobia and the fungal groups Ascomycota, Basidiomycota and Chytridiomycota. Among them, the bacteria genera of *Pseudonocardia* and *Rubrobacter* and unclassified fungi in the order of Capnodiales were dominant. The relative abundance of *Pseudonocardia* in the painted layer samples was higher than that in the dust sample, while Cyanobacteria dominated in the dust sample. Many of them have been discovered at other cultural heritage sites and associated with the biodeterioration of cultural relics. The presence and activity of these pioneering microorganisms may lead to an unexpected deterioration of the painted sculptures that are preserved in this heritage site. Thus, proper management strategies and potential risk monitoring should be used in the Maijishan Grottoes to improve the conservation of these precious painted sculptures.

## Introduction

The biodeterioration of cultural heritage is a ubiquitous and inevitable phenomenon that has attracted conservators, archaeologists and scientists who are involved in the conservation of cultural heritage and relics. Microorganisms are the most diverse group of known life in the world; they occupy almost all the niches of the biosphere due to their abundant diversity, tenacious vitality and exuberant metabolism. Previously, multiple reports have documented that almost all types of historic artifacts, including the mural paintings in temples, caves and catacombs [1–6], and even the art made of modern materials [7,8], exhibited signs of biodeterioration because of colonization and the microbes’ powerful biodeteriorative potential. Based on culture-dependent method, some representative functional microbial groups have been isolated from the wall paintings that are suffering from the deterioration, and abundant microbial diversity has also been detected in different niches using molecular identification methods [9–11]. Although previous studies were biased towards incompleteness due to their employed methods, core and common metabolically active microbes have been associated with biodeterioration in caves and similar niches [12,13]. Many previous studies have suggested that these microbes contribute to the deterioration process, but their roles and detailed mechanisms have not been ascertained due to a lack of research methods.

The selection of an appropriate strategy for understanding microfloral characteristics is indispensable for clarifying the ‘disease’ mechanisms induced by these microbes. Historically, microbial communities were mainly investigated by a culture-based method. However, only less than 1% of the microorganisms present in environmental samples can be cultured in laboratory conditions, and these cultures only represent the isolation of easily cultivable microorganisms [14]. Even so, culture-based methods still deserve recognition for the information derived from such procedures, as the isolated and purified strains can be used to further study the associated biochemical processes and mechanisms and their roles in specific environmental conditions and material types. Currently, multiple molecular approaches, such as DGGE, RFLP, FISH and qPCR, have been used to identify the presence of environmental microorganisms, especially non-culturable ones, to advance our understanding of the microflora on these treasured cultural sites and relics. Although molecular strategies are powerful tools for revealing microbial communities, including metabolic and destructive microorganisms, the weaknesses of culture-based methods [15,16] are their high cost, time-consuming data processing and low-throughput methods that cannot detect many taxa; these are apparent obstacles that must be overcome. Fortunately, the advances in high-throughput sequencing methods can overcome these problems and provide further insights into the microbial world.

In this study, the Maijishan Grottoes, a famous Buddhist cave temple located on the ancient Silk Road in China, was selected. The Maijishan Grottoes are known for their beautiful scenery, numerous caves, precious artwork and long-spanning history. The site was added to the World Heritage List in 2014 as one of the key historical sites of the “Silk Roads, the Route Network of the Chang'an-Tianshan Corridor.” The priceless painted sculptures that are preserved in the caves of the Maijishan Grottoes are severely threatened by multiple factors, including the poor stability of the mountain rocks, frequent earthquakes, water seepage, microclimate change and human disturbance, in addition to microorganisms. More severely, the cultural relics preserved in caves had been exposed to high humidity (RH ≥70%) in the atmosphere for long periods, namely, more than six months of the year (monitoring data, not shown). Generally, the physical conditions are considered extremely unfavorable to the conservation of various types of cultural relics [17–19]. Consequently, numerous painted sculptures showed visible signs of damage, such as cracking, powdering, salt crystallization and surface color changes, which appeared as fading or black spots, due to the adverse environmental conditions. Similar diseases were also a prevalent phenomenon observed at other heritage sites (e.g., the Mogao Grottoes) along the Silk Road in West China. Some of the artwork was severely damaged by the extensive growth of microorganisms that colonized on the wall paintings, and then this artwork was further damaged by the physical and chemical invasions from the biochemical and physiological activity of the colonizing microorganisms. Unlike other diseases, the accumulative process of microbial-induced biodeterioration can be extremely slow, and its symptoms are invisible in the early stages due to a lack of proper research experience. It is generally agreed upon that microorganisms cause serious contamination of the mural paintings and that they colonize and penetrate into the deep layers of the paintings, resulting in material loss due to acid corrosion, enzymatic degradation and mechanical attack [20,21].

Previously, research on the microbial invasions of wall paintings was only carried out at the Mogao Grottoes, a cultural heritage site along the Silk Road. As early as the 1990s, the staff of the Dunhuang Academy first isolated cultivable microorganisms from 51 samples of discolored paintings that were collected from six ancient caves constructed in different dynasties. Several functional genera, including *Aspergillus*, *Cladosporium* and *Flavobacterium* [22], were identified, and these microbes were associated with the accelerated degradation process of ancient wall paintings. Subsequently, simulated experiments revealed that some microbial metabolites, including the pigments and calcium oxalate, altered the shape of the pigment crystals and even decreased the crystals’ structural stability, as well as changing the elemental valence state of the chemical pigments [23]. These processes played a critical role in the deterioration and discoloration of the mural paintings, particularly for a red pigment containing lead red (Lead tetroxide, Pb_3_O_4_). Recently, the microfloral characteristics that were present on the wall paintings with signs of damage, along with their temporal and spatial distribution patterns in different caves, were also investigated. Furthermore, the key environmental parameters that influence community distribution patterns and their relationship with the ambient conditions were further investigated [5]. In addition, the phylogenetic composition of airborne microbes and their seasonal dynamics were investigated. Climatic parameters and tourist activities were regarded as the two main factors that contributed to the risk of biodeterioration [24,25]. The microbial species with invasion potential that could damage the wall paintings or act as opportunistic pathogens to the visitors were also identified [26,27]. Overall, these studies provided a comprehensive understanding of the microbes associated with mural decay in the Mogao Grottoes.

A comprehensive understanding of the microflora colonized on the painted sculptures in the Maijishan Grottoes is a crucial step for the effective protection and management of this site’s ancient relics in the future. In recent years, a huge number of tourists have been attracted by the Maijishan Grottoes. Statistically, approximately 57,000 visitors entered the grottoes during the May Day Holiday (1 May to 3 May) in 2016, but the maximum tourist capacity at the site is under 6,400 per day. Undoubtedly, too many tourists flooded into a relatively limited space in a short time, resulting in a micro-climate alteration, which, in many cases, is harmful to the conservation of cultural heritage sites. For example, the Paleolithic rock paintings of the Lascaux Cave suffered successive biological invasions since its discovery due to artificial disturbances, most of which were tourists [9,28]. Thus, the relationship between the conservation of heritage sites and tourism activities has become a hot topic in academic studies.

Overall, to improve the conservation of the cultural heritage preserved in the Maijishan Grottoes, it was necessary to investigate the microbial communities on the painted sculptures. We adopted an innovative molecular strategy, namely, Illumina MiSeq sequencing, to analyze the samples of the discolored painted sculptures that were collected from Cave 4–4 of the Maijishan Grottoes. Our research objectives were to: (1) describe the complete bacterial and fungal communities on the painted sculptures in the Maijishan Grottoes; (2) define the core phylogenetic groups among the whole microbial community; and (3) assess the potential threat of invasive microbial groups at this study site. This is the first report investigating the characteristics of the microbial community on the painted sculptures of the Maijishan Grottoes. It may not only contribute to clarifying mechanisms of biodeterioration induced by the inhabiting microbes but also provide opportunities for microbiologists to formulate preventive and remedial strategies to control the undesirable growth of microorganisms on this priceless and fragile artwork.

## Materials and methods

### Sampling sites and sampling process

**Fig 1:**
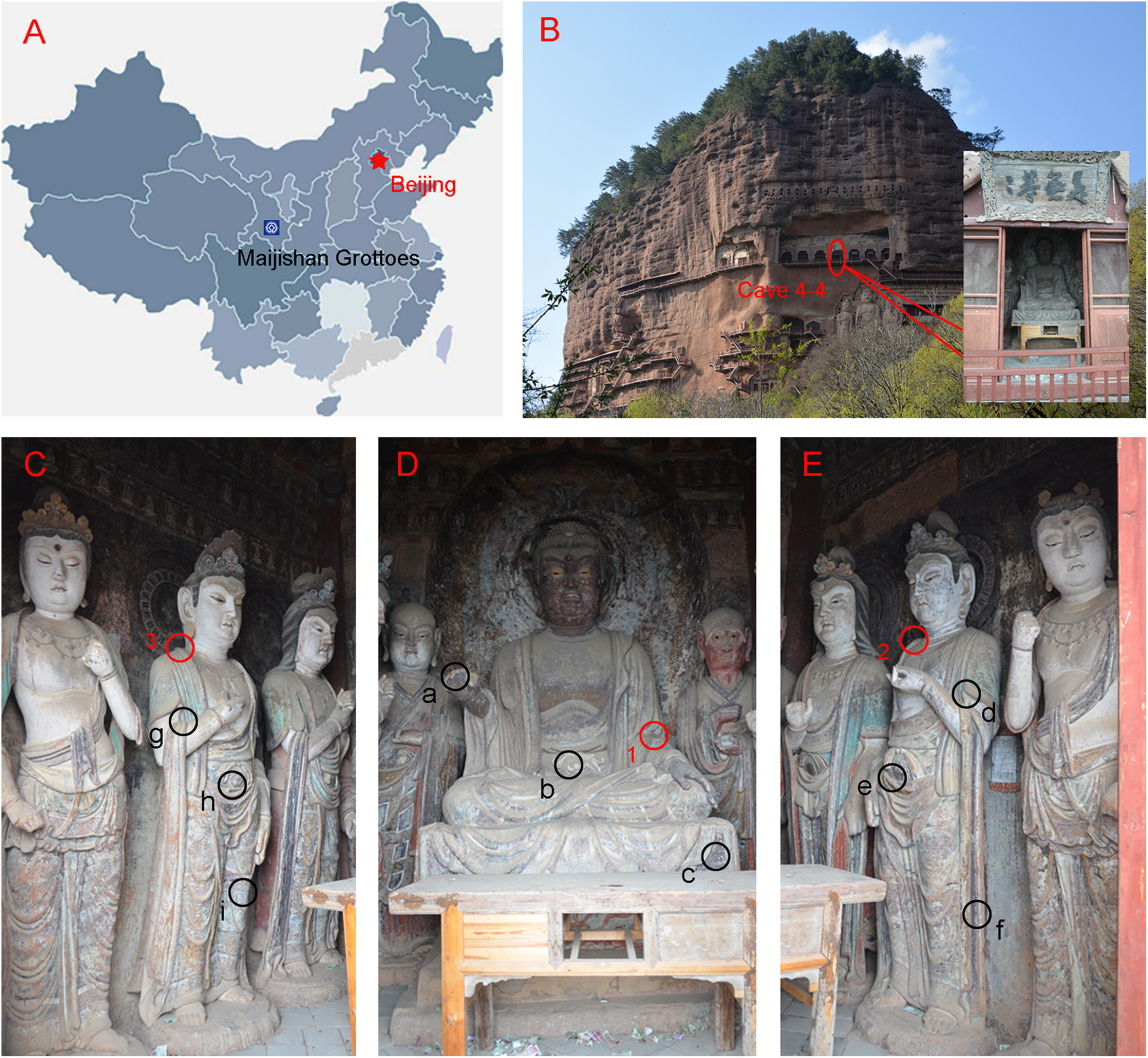
The research site and the sampling locations in the grottoes. A: The geographical location of the Maijishan Grottoes in China; B: Cave 4–4 of the Maijishan Grottoes; C, D and E: the painted statues inside Cave 4–4 and the sampling locations, samples a-c were collected from the Shakyamuni sculpture on the east side, were mixed thoroughly and were named it as MJ4-1. Similarly, samples d-f and g-I were collected from the Bodhisattva sculpture on the south and north sides, respectively. They were also pooled together and were named MJ4-2 and MJ4-3, respectively. Samples 1, 2 and 3 were mixed and were named MJ4-4.

The Maijishan Grottoes are situated in Maijishan, 30 km southeast of Tianshui city in the Gansu Province, China (Fig 1A). This city is located in the eastern Gansu Province, adjacent to the Shaanxi Province. The Silk Road is over 1600 kilometers in length within the Gansu Province, and Tianshui City was historically the first strategic location outside of Chang’an City (now called Xi’an, the capital of the Shaanxi Province) to the west; it was the capital of the ancient Chinese Kingdom and has played a very important role in the economic and cultural exchange between ancient China and the Western world.

The Maijishan Grottoes are surrounded by the Qinling Mountains. The Grottos have a warm, semi-humid continental climate with an average annual temperature of 10.4°C, an average annual relative humidity of 69.2% and an annual rainfall of 680 mm. A unique geographical advantage gives the location a pleasant climate, without brutal heat in the summer or bitter cold in the winter, and a clear rhythm of the four seasons, with a beautiful landscape and various well-known natural sites.

Three painted sculptures were selected for sampling in Cave 4–4 of the Maijishan Grottoes (N34°21.093′, E106°00.261′), the so-called “Upper Seven Buddha Pavilion,” which was built during Northern Zhou (557-581AD). For each statue, three independent sampling locations on the surface of painted layer were chosen, and the samples were carefully collected with sterile swabs and scalpels. Then, the samples were pooled together in three parallel repetitions and mixed thoroughly to form a single sample (Fig 1C, 1D and 1E). Only a tiny amount of the painted layers was allowed to be collected during the sampling process, and the sampling locations were restricted to the edges of the damaged paintings. For the material specificity of the painted sculptures, a single sample was not sufficient for MiSeq sequencing. Thus, in this study, three single samples from each statue were mixed together as one representative sample per statue and were named MJ4-1, MJ4-2 and MJ4-3. At the same time, the floating dust deposited on the surface of the painted sculptures was collected as a control. One sampling location was identified for dust collection on each statue, and then the samples were mixed together as a single sample, called MJ4-4.

All the samples were collected under strict aseptic conditions. The sample materials were collected in a sterile centrifuge tube and stored at -20°C until further analysis. The sampling process was authorized and supervised by the administrative staff of the Art Institute of the Maijishan Grottoes.

### MiSeq high-throughput sequencing

The four sample materials were used for genomic DNA extraction with a commercially available extraction Kit (OMEGA Laboratories Inc., USA) according to the manufacturer’s protocols. For each sample, the integrity of the DNA was determined by electrophoresis on 1.0% agarose gels, and the concentration and purity of the DNA were measured spectrophotometrically with a NanoDrop^®^ ND-2000 (Thermo Scientific Inc., USA).

The universal primers 338F/806R for bacteria or 817F/1196R for fungi was used for the amplification and MiSeq sequencing of the PCR products. The 16S rRNA genes were amplified using the primer set: 338F, 5'-ACTCCTACGGGAGGCAGCAG-3', and 806R, 5'-GGACTACHVGGGTW TCTAAT-3' [29], which target the V3-V4 regions of the 16S rRNA genes. The 18S rRNA genes were amplified with the primer set: 817F, 5'-TTAGCATGGAATAATRRAATAGGA-3', and 1196R, 5'-TCT GGACCTGGTGAGTTTCC-3' [30], which target the V5-V7 regions of the 18S rRNA genes.

To minimize the impact of potential early round PCR errors, PCR was carried out in triplicate with a 20-μL reaction volume, containing 4 μL of 5-fold reaction buffer, 4 μL of dNTPs (2.5 mM), 0.8 μL of each primer (5 μM), 0.2 μL of BSA (20 μg/μL), 1 μL (approximately 10 ng) of template DNA and 0.4 μL of Pfu DNA Polymerase (TransStart-FastPfu DNA Polymerase, TransGen Biotech), with dd H_2_O to the final volume. The PCR program included an initial denaturation at 95°C for 3 min; 35 cycles at 94°C for 30 s, annealing at 55°C for 30 s and an extension at 70°C for 45 s; and a final extension step at 72°C for 10 min. PCR was performed using an ABI GeneAmp^®^ 9700 Cycler (Thermo Scientific Inc., USA). Different “barcode” sequences were added at the 5*'* end of the forward primer to separate the corresponding reads from the data pool that was generated in a single sequencing run. The amplicons were extracted by electrophoresis in 2.0% agarose gels, purified using a Gel Extraction Kit (AXYGEN Co., China) according to manufacturer’s instructions and quantified using a QuantiFluor™-ST Fluorimeter (Promega, USA). The purified amplicons were pooled in an equimolar and paired-end sequence (2×300) on an Illumina MiSeq PE300 Sequencer (Majorbio Co. Ltd., Shanghai) according to standard protocols.

### Processing and analysis of the sequencing data

Raw FASTQ files were de-multiplexed and quality-filtered using Trimmomatic (Version 0.35) with the following criteria: (ⅰ) The 300-bp reads were truncated at any site that obtained an average quality score below 20 over a 50-bp sliding window, and the truncated reads shorter than 50 bp were discarded; (ⅱ) Extracted matching barcodes, two nucleotide mismatch in primer matching, and reads containing ambiguous characters were removed; (ⅲ) Only overlapping sequences longer than 10 bp were assembled according to their overlapping sequence. Reads that could not be assembled were discarded. Quality sequences were aligned in accordance with SILVA alignment [31] and clustered into OTUs using USEARCH version 7.1. Operational taxonomic units (OTUs) with a 97% similarity level were used for the Rarefaction curve, and an α-diversity index including Ace, Chao, Shannon diversity, Simpson diversity indices and Coverage analysis were used with MOTHUR (1.30.1) [32]. Taxonomical assignments of the OTUs at 97% similarity were performed using MOTHUR in accordance with SILVA (123) or Unite (7.0) at 70% confidence intervals. For taxonomic analysis, the SILVA database (http://www.arb-silva.de) and the Unite database (http://unite.ut.ee/index.php) were used for bacteria and fungi, respectively. The β-diversity analysis, including Principal Component Analysis (PCA) and a hierarchical heatmap, was generated using the Vegan packages in R (3.2.0), while the Venn diagrams were implemented by the Venn diagram package. The raw reads were deposited into the NCBI Sequence Read Archive (SRA) database (SRA accession: SRP095989).

## Results

### MiSeq sequencing results and α-diversity indices

From the four samples, 133,014 valid reads and 1,757 OTUs were obtained for bacteria. Each library contained 21,369 to 38,038 reads with different phylogenetic OTUs, ranging from 232 to 870. The average length of the quality sequences was 434 to 441 bp. In addition, 101,015 valid reads and 217 OTUs were found for fungi, and the average length of the quality sequences was approximately 403 bp. The total number of sequences, coverage, number of OTUs, and statistical estimates of the species richness for 15,595 sequences (bacteria) or 18,193 sequences (fungi), together with the subsets from each sample at a genetic distance of 3%, are presented in S1 Table and S2 Table. The rarefaction curves tended to approach the saturation plateau in all samples (S1 Fig), indicating that the data volume of obtained sequences was reasonable, and an increase in the number of reads may contribute a slight increase in the total number of OTUs. The curves show that only a very small fraction of the new phylotypes of the microorganisms was retrieved after 15,000 sequencing reads.

For bacteria, MJ4-4 had the highest richness (Ace = 1059, Chao = 1039), while MJ4-1 had the lowest (Ace = 546, Chao = 367). The Shannon index provides not only the simple species richness (the number of species present) but also the abundance of each species (the evenness of the species) as distributed among all the species in the community. MJ4-4 had the highest diversity (Shannon = 5.12), which was much larger than the diversity of MJ4-3 (Shannon = 3.42) among the four communities, and MJ4-1 had the lowest diversity (Shannon = 2.15). A total of 232 to 870 OTUs were achieved in the whole samples at a 3% distance. The rarefaction curve progression (98.60–99.39%, Good’s Coverage) was very close in all the samples. The Simpson index varied from 0.0208 to 0.2665, with the highest value for MJ4-4 and lowest value for MJ4-1 (S1 Table).

For fungi, MJ4-4 also had the highest richness (Ace = 72, Chao = 73), followed by MJ4-3 (Ace = 71, Chao = 70), and MJ4-1 had the lowest richness (Ace = 45, Chao = 44). MJ4-3 had the highest diversity (Shannon = 2.41) whereas MJ4-2 had the lowest diversity (Shannon = 1.72). The number of OTUs ranged from 42 to 67 in these samples; MJ4-4 had the highest one and MJ4-1 had the lowest one (S2 Table).

### Taxonomic composition based on MiSeq sequencing

**Fig 2:**
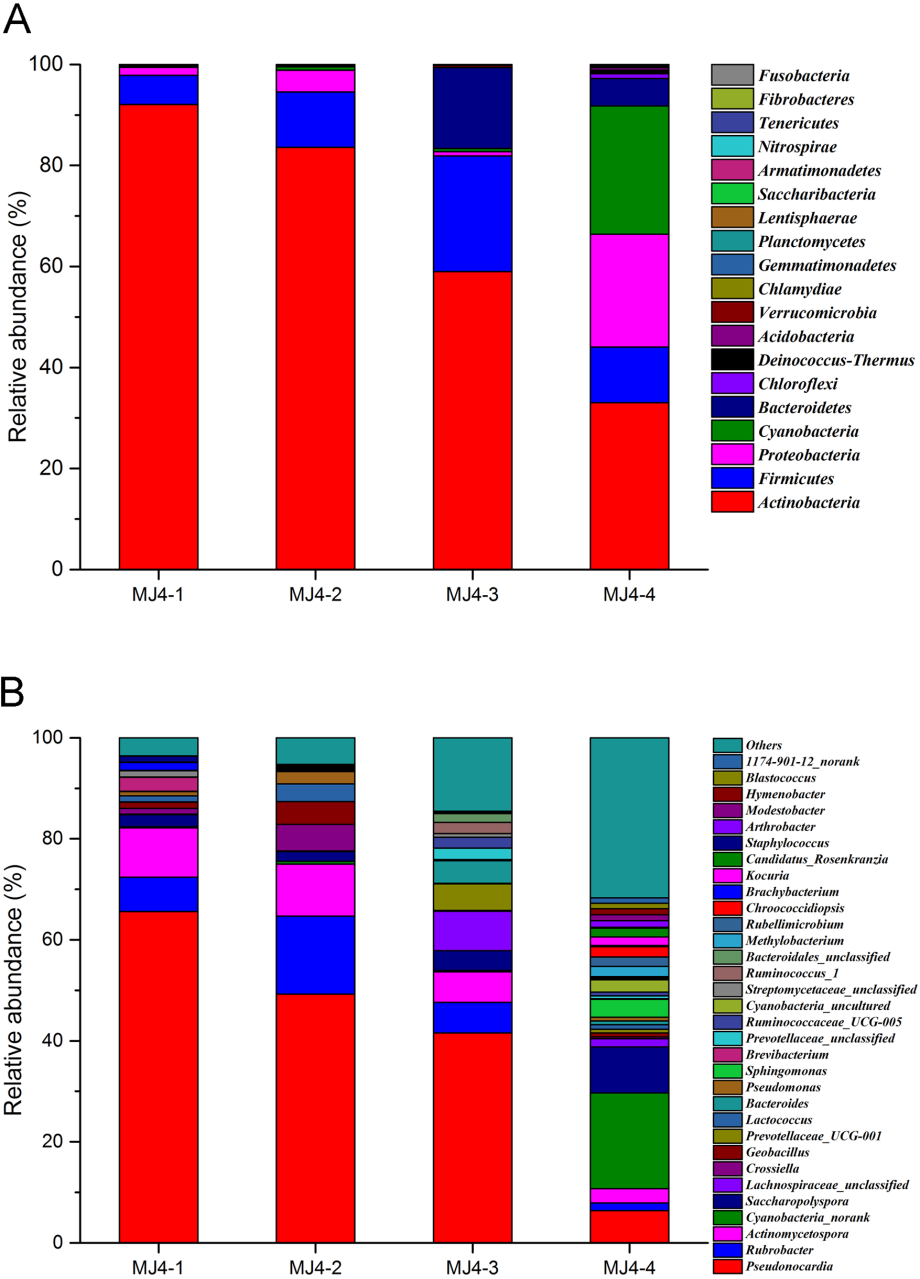
Relative abundance of the bacterial community at the phylum (A) and genus (B) level.

The bacterial OTUs can be assigned into 19 phyla, 202 families and 435 genera. Eight different phyla, namely, Actinobacteria, Acidobacteria, Bacteroidetes, Cyanobacteria, Chloroflexi, Firmicutes, Proteobacteria and Verrucomicrobia, out of the 19 total bacterial phylotypes were common to the four libraries (Fig 2), contributing to 99.96%, 99.93%, 99.82% and 98.88% of the total reads in the libraries of MJ4-1, MJ4-2, MJ4-3 and MJ4-4, respectively. Actinobacteria was the most abundant division, comprising 20.88% (367) of the OTUs and 53.53% (41,741) of the reads across all samples. Firmicutes, the second most abundant phylum, was 28.45% (500) of the OTUs and 12.79% (7,911) of the reads in all libraries. These two phyla collectively accounted for 97.86%, 94.56% and 81.9% of the total reads in MJ4-1, MJ4-2 and MJ4-3, respectively (Fig 2A). However, Actinobacteria showed a high variability in the read abundance of different samples; in decreasing order of abundance, the results are MJ4-1 (92.08%, 14,361 reads), MJ4-2 (83.56%, 13,032 reads), MJ4-3 (58.99%, 9,201 reads) and MJ4-4 (33.0%, 5,147 reads). Meanwhile, the reads of Firmicutes fluctuated in the different samples; the read proportion reached the lowest value in MJ4-1, and it was the fourth largest phylum (11.04%, 1,723 reads) in MJ4-4, which occurred after Cyanobacteria (25.45%, 3,969 reads) and Proteobacteria (22.31%, 3479 reads). The members of Acidobacteria, Bacteroidetes, Chloroflexi, Cyanobacteria and Proteobacteria comprised 0.16% (99 reads), 5.45% (3,402 reads), 0.27% (171 reads), 6.76% (4,218 reads) and 7.25% (4,521 reads), respectively, in the total bacterial community. The other lineages represented a much smaller fraction (ca 4.10%, 72 OTUs; 0.5%, 317 reads) of the bacterial community.

At the genus level, the genera of *Pseudonocardia* and *Saccharopolyspora* accounted for only 5.41% of the total OTUs, but 54.06% of the total reads (18.80–78.90%)(Fig 2B). *Rubrobacter* contained only 17 OTUs, with an average of 7.47% of the total reads with a high variability (1.53–15.46%). The subgroups of Micrococcaceae, Brevibacteriaceae, Geodermatophilaceae, Streptomycetaceae, Dermabacteraceae and Propionibacteriaceae were less than 1% of the total reads, accounting for 0.92%, 0.76%, 0.65%, 0.64%, 0.50% and 0.33%, respectively. A low relative abundance of *Pseudonocardia* (6.35%) was found in MJ4-4 compared to the other three libraries (41.54–65.57%) of this study. In contrast, MJ4-4 had a higher abundance of Cyanobacteria without rank (18.92%) than the other three samples (0.23–0.56%).

The fungal communities were assigned to 6 phyla, 53 families and 46 genera. Ascomycota was the most dominant division, comprising 67.74% (147) of the OTUs and 94.73% of the total reads. Basidiomycota was the second largest division, with 28.57% (62) of the OTUs and 5.17% of the total reads; the other four fungal phyla, namely, Chytridiomycota, Ciliophora, Eukaryota_unclassified and Phragmoplastophyta, accounted for only 0.1%. The sequences affiliated with the Ciliophora were non-specific amplification products, and the application of universal eukaryotic primers resulted in the sequences of some Eukaryota that could not be classified.

**Fig 3:**
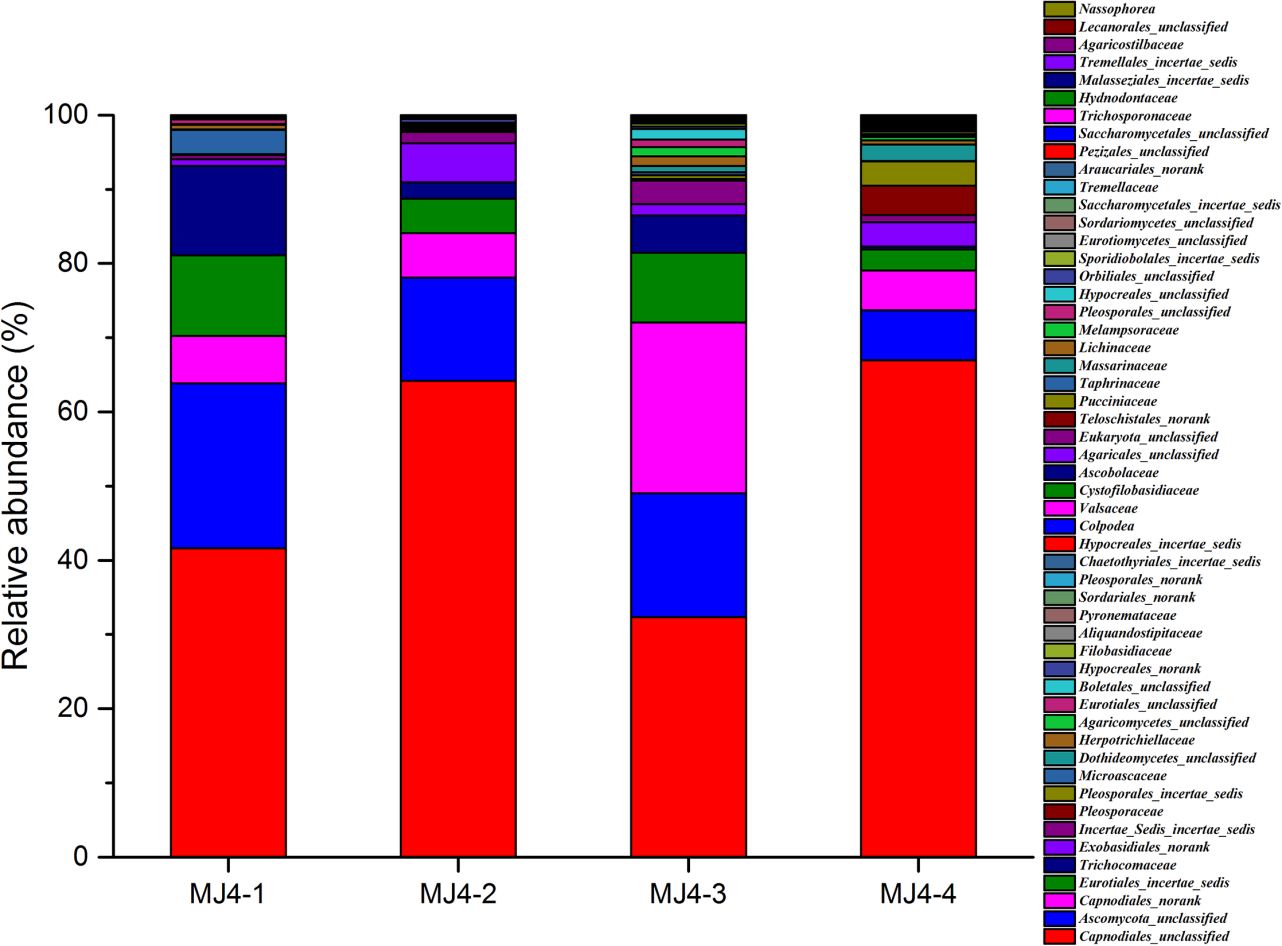
Relative abundance of the fungal community at the family level.

At the family level, abundant unclassified or no rank fungal sequences were found in each sample. The total relative abundances of the unclassified and no rank fungi in MJ4-1, MJ4-2, MJ4-3 and MJ4-4 were 83.93%, 97.23%, 92.15% and 93.71%, respectively (Fig 3). The members of Capnodiales dominated the Ascomycota phylum, represented by Capnodiales_unclassified and Capnodiales_no rank, and accounted for approximately 51.28% (37,319) and 10.19% (7,421) of the reads across all samples, respectively. Ascomycota_unclassified, the second most abundant group, (11.98%, 26 OTUs) consisted of 14.88% (10,832) of the reads in all libraries. However, the proportion of Capnodiales_unclassified in different samples showed a high variability, with a range of 32.36% -66.97%. The proportion of reads was the lowest in MJ4-3, while it was the highest in MJ4-4, followed closely by MJ4-2 (64.17%). Meanwhile, the relative abundance of Ascomycota_unclassified showed an increasing trend in three painted layers compared to MJ4-4, ranging from 6.71% to 22.25%, 13.89% and 16.17%, respectively. These three families, including Capnodiales_unclassified, Ascomycota_unclassified and Capnodiales_no rank, were more than 76.3% of the total sequences in all four samples. A low relative abundance of Eurotiales (2.94%), represented by Eurotiales_incertae_ sedis and Eukaryota_unclassified, was detected in MJ4-4 compared to the other three libraries.

### Microbial community structure among different samples based on MiSeq sequencing

**Fig 4:**
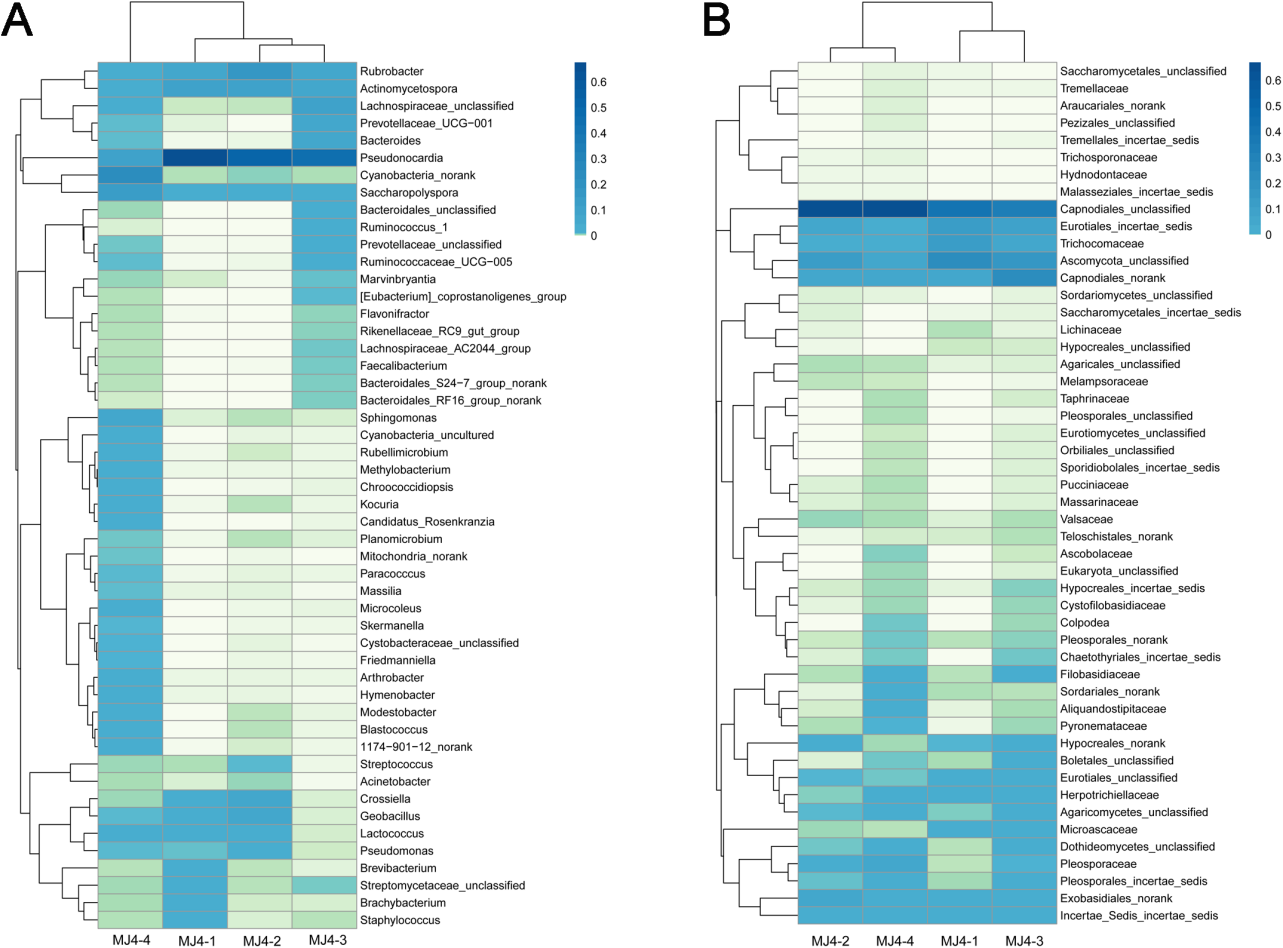
Heat map representation and cluster analysis of the microbial community among four samples. Bacterial (A) and fungal (B) distributions of the top 50 abundant genera and families, respectively. The double hierarchical dendrogram shows the bacterial and fungal distribution. The bacterial and fungal phylogenetic trees were calculated using the neighbor-joining method.

**Fig 5:**
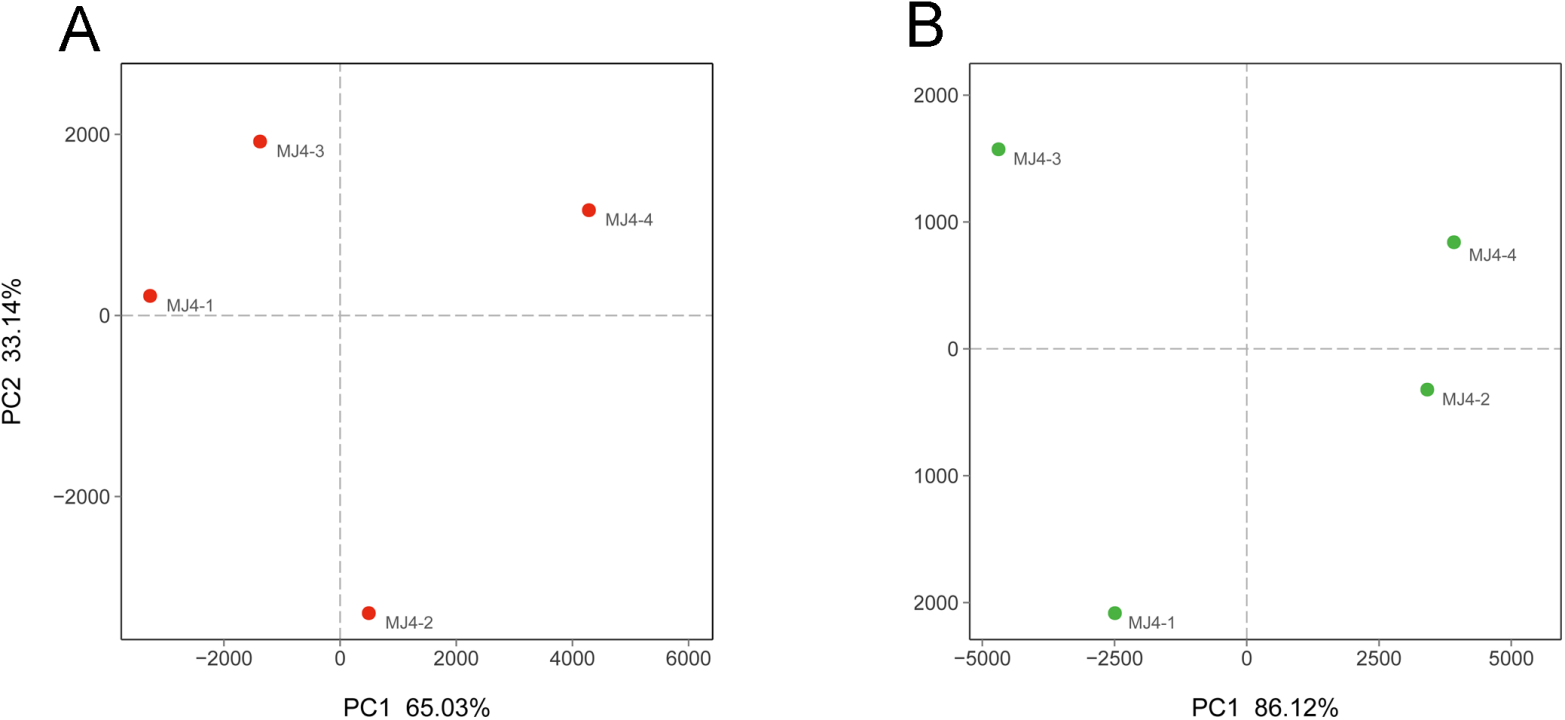
Principal components analysis. Scatter plot of the PCA-score showing the similarity of the bacterial (A) and fungal (B) communities based on the Unifrac distance.

To analyze the similarity of the microbial communities among the samples, a heatmap was conducted using hierarchical cluster analysis. For bacteria, the heatmap (Fig 4A) was based on the top 50 abundant bacterial genera and showed that MJ4-2 and MJ4-3 were grouped together first, then clustered with MJ4-1; MJ4-4 was significantly different from the other three samples. The PCA results (Fig 5A) also revealed that the bacterial communities of MJ4-1 and MJ4-3 grouped to the left of the graph along PC1, which accounts for 65.03% of the total variations, whereas MJ4-2 was separate from other samples along PC2, which represented 33.14% of the total variations.

**Fig 6:**
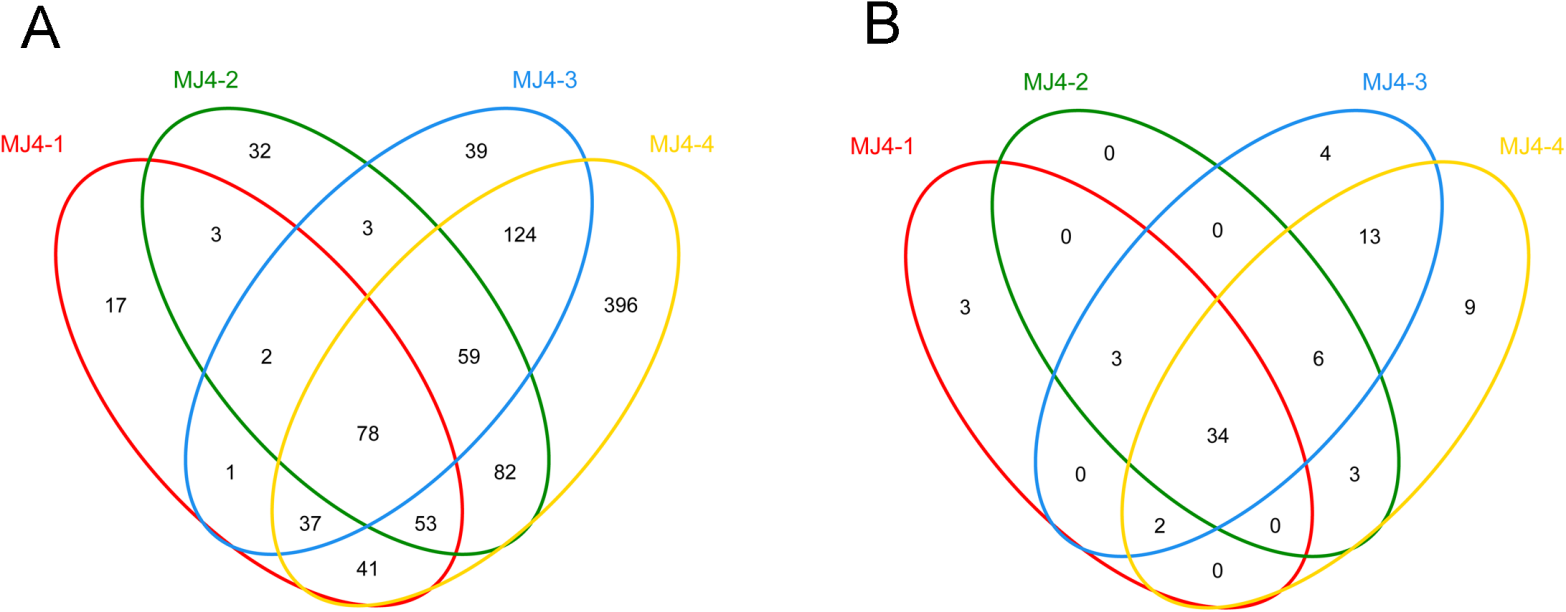
Shared OTU analysis of the different samples. Venn diagram showing the unique and shared OTUs (97%) for the bacterial (A) and fungal (B) communities among the four samples.

The species shared among the bacterial communities were further determined via a Venn diagram to compare the relationships among these samples in more detail. The results showed that a total of 78 shared OTUs were evident in all the samples (Fig 6A), which comprised 97.04%, 95.36%, 63.18% and 49.12% of reads in MJ4-1, MJ4-2, MJ4-3 and MJ4-4, respectively. Actinobacteria and Firmicutes dominated in the shared OTUs (S3 Table), as well as the reads of the shared OTUs. Out of the 968 OTUs in total, three libraries from MJ4-1, MJ4-2 and MJ4-3 had 80 OTUs in common, which comprised 97.17%, 95.4% and 63.19% of the quality reads in each library, respectively. The core microbiotas were dominated by Actinobacteria and Firmicutes, which are represented by Pseudonocardiaceae, Rubrobacteriaceae, Bacillaceae and Streptococcaceae. The members of *Pseudonocardia* dominated the family of Pseudonocardiaceae (S5 Table) with only 7 OTUs, but they were more than 75.37% (25,293) of the total reads across all the samples. In addition, a total of 396 OTUs were observed in only the MJ4-4 library. These unique OTUs comprised 11.84% (1,847) of the total reads in the MJ4-4 library and were dominated by Proteobacteria (107 OTUs, 486 reads) and Actinobacteria (98 OTUs, 364 reads).

For fungi, the heatmap (Fig 4B) was based on the top 50 fungal families. The figures show that the samples divided into two clusters at the family level: MJ4-1 and MJ4-3 into one group, and MJ4-2 and MJ4-4 into another. The PCA score plot (Fig 5B) agrees with the heatmap, indicating a high similarity of the fungal communities between MJ4-1 and MJ4-3 and between MJ4-2 and MJ4-4. Clearly, MJ4-1 and MJ4-3, in addition to MJ4-2 and MJ4-4, grouped to the left or the right of the graph along the PC1 axis, which accounts for 86.12% of the total variations. The PC2 axis explained only 11.89% of the variance.

The Venn diagram shows that the four libraries own 34 fungal OTUs in common (Fig 6B), accounting for 99.86%, 99.84%, 99.13% and 98.77% of the reads in MJ4-1, MJ4-2, MJ4-3 and MJ4-4, respectively. Ascomycota dominated in the shared OTUs as well as the reads of the shared OTUs (S4 Table, S6 Table), and the rest of the shared OTUs belonged to Basidiomycota. Of the 77 total OTUs, the three libraries from MJ4-1, MJ4-2 and MJ4-3 had 37 OTUs in common. Actually, the OTUs shared by all four libraries were completely overlapped with the OTUs shared by these three samples, with the exception of only 3 OTUs. In addition, 9 OTUs were detected in only the MJ4-4 library; these unique OTUs comprised a tiny fraction of the reads in MJ4-4 (ca.0.10%), and their effects on the community composition of MJ4-4 would be negligible.

## Discussion

To investigate the microbial communities of the painted sculptures in the Maijishan Grottoes, a MiSeq high-throughput sequencing approach was used. The results revealed a high bacterial diversity and a relatively low fungal diversity, as well as a high bacterial abundance and a low fungal abundance, on the painted sculptures of the Maijishan Grottoes. Many of the identified taxonomical groups in our report have been discovered at other cultural heritage sites.

For bacteria, the results suggested that Actinobacteria and Firmicutes were the most predominant bacterial taxa on the painted sculptures in the Maijishan Grottoes. Actinobacteria are well known for their high secondary metabolism, such as the metabolism of pigments, organic acids, polysaccharides and potent antibiotics. Over the past decades, an abundance of Actinobacteria was recorded in many caves or subterranean environments [3,33–35], while Firmicutes was frequently associated with earthy and drought environments. In addition, Acidobacteria, Bacteroidetes, Cyanobacteria, Chloroflexi and Proteobacteria were also detected in caves and subterranean environments. Comparing the distribution pattern of the bacterial genera among these samples, *Pseudonocardia* accounted for the largest number of sequences detected from three painted samples, ranging from 41.54% to 65.57%; its proportion was far higher in the painted samples than in dust (6.35%), indicating a significant community succession on the painted sculptures in the Maijishan Grottoes over time. *Pseudonocardia* was discovered in many heritage sites, particularly in catacombs and subterranean caves. Subterranean caves are generally regarded as oligotrophic ecosystems with almost no natural light, little or no organic matter, high salinity and a relatively constant temperature and relative humidity, all of which represent extreme environmental conditions for most microorganisms [36].

Generally, the surface of the wall paintings was also an extreme environment for most of the surviving bacteria because of its poor nutrient availability and dry conditions. In the process of creating the painted sculptures in the Maijishan Grottoes, some of the pigments contained heavy metal compounds as main components, such as lead red (Pb_3_O_4_) and vermilion (HgS), which are both widely used red pigments at our study site; these pigments have a strong toxicity that inhibits the reproduction of the majority species in bacterial communities. Thus, most species of microbes cannot survive in this especially hostile microenvironment. However, *Pseudonocardia* is suitable for niches of poor nutrition because they can develop their filamentous growth and utilize a variety of nitrogen and carbon sources. Thus, *Pseudonocardia* colonized the wall paintings in caves at an early stage of microbial colonization, before the subsequent deterioration and damage to the substratum materials [37]. *Pseudonocardia* can alter the local nutritional conditions and pH in a manner that benefits other life forms, mostly fungi and algae. The common, core metabolically active group was responsible for the degradation and discoloration of the wall paintings in many caves [38–40]. Their proliferation not only leads to the formation of white colonies on the paintings due to the formation of slightly alkaline niches but also results in a material loss that is caused by mycelium penetrating deeply into the paint layer of materials, further altering the hostile niches in favor of the proliferation of other microbes in unexpected microbial outbreaks.

*Rubrobacter* was the second largest bacterial genus detected in our research. Previous studies noted that the *Rubrobacter*-related bacteria are associated with the typical rosy stains on wall paintings or plaster in Europe [41–43]. In addition, they were detected in salmon pink biofilms from the bas-relief wall of the Bayon temple in Cambodia’s Angkor Thom [44], and most of the 16S rRNA sequences retrieved from the different sample sites were slightly separated from each other. Curiously, species of *Rubrobacter*-related bacteria were rarely confirmed, as most of them were detected in halophilic environments, especially in salt-attacked monuments [45]. The formation of typical rosy discolorations was associated with carotenoid pigments, such as β-carotene, α-bacterioruberin and derivatives from the secondary metabolism of *Rubrobacter*-related bacteria, and it significantly decreased the aesthetic appearance of wall paintings and historical plaster. Thus, large amounts of *Pseudonocardia* and *Rubrobacter* were detected on the painted sculptures in the Maijishan Grottoes, and this result is a strong indication of the potential risk of microbial invasion and deterioration.

Many heterotrophic bacterial genera, such as *Arthrobacte*r, *Brevibacterium*, *Bacillus*, *Kocuria*, *Pseudomonas*, *Streptomyces* and *Saccharopolyspora* were also detected in our research; these are also the species most frequently isolated from the wall paintings of caves and catacombs [46–50]. They played significant roles in the biodeterioration occurring on the wall paintings. For example, *Pseudomonas*-related bacteria were responsible for the “second microbial crisis” due to their highly resistant to antimicrobial biocides in the Lascaux Cave [51]. Some strains of *Pseudomona*s-related bacteria, such as *P*. *geniculata*, *P*. *stutzer*i and *P*. *fluorescen*s, were involved in the process of nitrogen fixation, which provides inorganic N from ammonia-ammonium for the generation of nitrous (nitric) acid by other microorganisms, causing acid corrosion to wall paintings, marble and limestone [52]. Other bacterial genera, including *Arthrobacter*, *Bacillus* and *Kocuria*, were identified in diverse environments, such as soil, aerosols and ancient buildings [44,49,53] and were shown to contribute to the degradation of wall paintings and other artwork [2,54,55]. Interestingly, Cyanobacteria and Proteobacteria were dominant phyla in dust, second only to Actinobacteria, but their proportions on the painted sculptures were far below that in dust. Most sequences of Cyanobacteria belonged to unclassified and uncultured taxa, while *Sphingomonas*, *Pseudomonas* and *Enterobacter* were the representative genera in Proteobacteria. Clearly, the main reasons for this phenomenon were that these two phyla were inhibited on the painted sculptures due to the extreme environment and fierce competition from the other dominant genus, *Pseudonocardia*.

For fungi, the results showed that a large number of sequences belonged to the unclassified or no rank genera based on the current database of 18S rRNA gene sequences, and most of the sequences were affiliated with Capnodiales (unclassified and no rank) and Ascomycota_unclassified. The results revealed a weakness of the fungi primers 817F/1196R for MiSeq sequencing due to their narrow taxonomical variety coverage [30,56,57]. Of course, the limitation of the fungal database was also important reason for this phenomenon.

Comparing the microfloral structures on the painted layers and in the deposited dust, we found that the diversity and abundance of bacteria were far higher in the dust than on the painted layers. Apart from 78 shared OTUs detected in all samples, a total of 396 bacterial phylogenetic OTUs were observed in the dust sample only. In addition, there were slightly more fungal phylogenetic OTUs in the dust than the number shared among all four libraries. In other words, almost all of bacteria and fungi detected on the painted layers can be found in the deposited dust, which was derived from the surrounding environment. To a large extent, the microfloral characteristics of the deposited dust were highly consistent with the surrounding atmosphere of the painted sculptures, suggesting that the air is an important source of microbes for painted sculptures.

In recent years, the Maijishan Grottoes and its elegant artwork and beautiful scenery have attracted an increasing number of tourists, exerting tremendous pressure on the conservation and management of this site. Previous studies revealed that when masses of tourists suddenly swarm into a cave in a short time, they may destroy the original conservation environment of the cultural relics due to their disturbances, such as the resuspension and floating of dust, an increase in temperature, relative humidity and CO_2_ concentration and an increase in the diversity and abundance of airborne microorganisms [24,25]. These unexpected changes always have a strongly irreversible influence on the cave microbiology and ecosystem stability. In the Altamira cave, the temperature increased 0.07°C and the CO_2_ concentration rose 51 ppm after one group of visitors entered the cave; these changes could not be restored to the initial background levels immediately, and, sometimes, they could not even be restored by the next day. Finally, the cave was closed for an indefinite period in 2002 due to the serious microbial outbreaks that were mostly caused by human disturbance [58,59]. Furthermore, tourists carried exogenous microbes into the caves and posed an increasing potential risk of pathogens. Some of the microbes, such as *Pseudomonas*, *Brevibacterium*, *Sphingomonas* and *Staphylococcus*, are pathogenic to humans and cause a potential danger to tourists and managers, but their existence and infection potential in humans are currently not understood [26,27,60]. Thus, complex management issues at the heritage site should take into account the visitor cycle and its frequency, and they should monitor the physical environmental conditions and the microorganisms in the dispersion process and colonization strategies. Overall, the best way to conserve and protect a cultural heritage site is to avoid human intervention to maintain the sophisticated ecosystem balance in the caves.

In summary, the characterization of the microbial communities on the painted sculptures in the Maijishan Grottoes showed the presence of complex bacterial and fungal groups on these painted sculptures, many of which are common core species associated with the biodeterioration of painted sculptures. In many cases, tiny fractions of microorganisms may result in severe damage to wall paintings due to a lack of sufficient attention or inappropriate management. Although no obvious visible microbial damage exists on the painted sculptures in the Maijishan Grottoes, the priceless and fragile artwork is now under multiple threats by different microorganisms and human activities. Furthermore, the local environmental conditions are favorable for microbial growth and reproduction and are dangerous to the painted sculptures, particularly the high relative humidity and human disturbances, increasing the potential risk of microbial outbreaks [61]. Thus, more attention should be paid to the conservative management of microbial threats and risk monitoring in the Maijishan Grottoes for preventive heritage conservation.

## Supporting information

S1 Fig Rarefaction curves. Rarefaction curves show the number of reads with the number of phylotypes at 97% sequence similarity level for the different samples. A: Bacteria, B: Fungi.
(DOCX)

S1 Table Phylotype coverage and diversity estimation of the 16S rRNA gene libraries of the samples from MiSeq sequencing analysis. The operational taxonomic units (OTUs) were defined with 97% similarity threshold. The coverage percentages, richness estimators (ACE and Chao), and diversity indices (Shannon and Simpson) were calculated.
(DOCX)

S2 Table Phylotype coverage and diversity estimation of the 18S rRNA gene libraries of the samples from MiSeq sequencing analysis. The operational taxonomic units (OTUs) were defined with 97% similarity threshold. The coverage percentages, richness estimators (ACE and Chao), and diversity indices (Shannon and Simpson) were calculated.
(DOCX)

S3 Table The shared phyla among the 16S rRNA gene libraries from the four mixed samples. (DOCX)

S4 Table The shared phyla among the 18S rRNA gene libraries from the four mixed samples. (DOCX)

S5 Table The reads of the predominant bacterial OTUs in the four mixed samples. Eighteen OTUs with abundances higher than 0.5% in the bacterial community were sorted from total of 1,757 OTUs, and defined as predominant OTUs. S: = species; G: = genus; F: = family; O: = order; C: = class; P: = phylum.
(DOCX)

S6 Table The reads of the predominant fungal OTUs in the four mixed samples. Fifteen OTUs with abundances higher than 0.5% in the fungal community were sorted from total of 217 OTUs, and defined as predominant OTUs. S: = species; G: = genus; F: = family; O: = order; C: = class; P: = phylum.
(DOCX)

## References

[1] Pepe O, Sannino L, Palomba S, Anastasio M, Blaiotta G, Villani F, et al Heterotrophic microorganisms in deteriorated medieval wall paintings in southern Italian churches. Microbiol Res. 2010; 165(1):21–32. doi: 10.1016/j.micres.2008.03.005 18534834 10.1016/j.micres.2008.03.005

[2] Capodicasa S, Fedi S, Porcelli AM, Zannoni D. The microbial community dwelling on a biodeteriorated 16th century painting. Int Biodeter Biodegr. 2010; 64(8):727–733.

[3] Cuezva S, Fernandez-Cortes A, Porca E, Pašić L, Jurado V, Hernandez-Marine M, et al The biogeochemical role of Actinobacteria in Altamira Cave, Spain. FEMS Microbiol Ecol. 2012; 81(1): 281–290. doi: 10.1111/j.1574-6941.2012.01391.x 22500975 10.1111/j.1574-6941.2012.01391.x

[4] Zucconi L, Gagliardi M, Isola D, Onofri S, Andaloro MC, Pelosi C, et al Biodeterioration agents dwelling in or on the wall paintings of the Holy Saviour’s cave (Vallerano, Italy). Int Biodeter Biodegr. 2012; 70(6): 40–46.

[5] Ma YT, Zhang H, Du Y, Tian T, Xiang T, Liu XD, et al The community distribution of bacteria and fungi on ancient wall paintings of the Mogao Grottoes. Sci Rep. 2015; 5:7752 doi: 10.1038/srep07752 25583346 10.1038/srep07752 PMC4291566

[6] Lan WS, Li H, Wang WD, Katayama Y, Gu J-D. Microbial community analysis of fresh and old microbial biofilms on Bayon Temple Sandstone of Angkor Thom, Cambodia. Microb Ecol. 2010; 60(1):105–115. doi: 10.1007/s00248-010-9707-5 20593173 10.1007/s00248-010-9707-5 PMC2917545

[7] Sabev HA, Barratt SR, Handley PS, Greenhalgh M, Robson GD. Biodegradation and biodeterioration of man-made polymeric materials In: Gadd GM (Ed) Fungi in biogeochemical cycles. Cambridge University Press 2006.

[8] Gu J-D, Kigawa R, Sato Y, Katayama Y. Addressing the microbiological problems of cultural property and archive documents after earthquake and tsunami. Int Biodeter Biodegr. 2013; 85(7): 345–346.

[9] Jurado V, Fernandez-Cortes A, Cuezva S, Laiz L, Cañaveras JC, Sanchez-Moral S, et al The fungal colonisation of rock-art caves: experimental evidence. Sci Nat. 2009; 96(9): 1027–1034. 10.1007/s00114-009-0561-6 19484211

[10] Vasanthakumar A, De Araujo A, Mazurek J, Schilling M, Mitchell R. Microbiological survey for analysis of the brown spots on the walls of the tomb of King Tutankhamun. Int Biodeter Biodegr. 2013; 79(4): 56–63.

[11] Principi P, Villa F, Sorlini C, Cappitelli F. Molecular studies of microbial community structure on stained pages of Leonardo da Vinci’s Atlantic Codex. Microb Ecol. 2011; 61(1): 214–222. doi: 10.1007/s00248-010-9741-3 20811884 10.1007/s00248-010-9741-3

[12] Porca E, Jurado V, Žgur-Bertok D, Saiz-Jimenez C, Pašić L. Comparative analysis of yellow microbial communities growing on the walls of geographically distinct caves indicates a common core of microorganisms involved in their formation. FEMS Microbiol Ecol. 2012; 81(1): 255–266. doi: 10.1111/j.1574-6941.2012.01383.x 22486654 10.1111/j.1574-6941.2012.01383.x

[13] Saiz-jimenez C. Microbiological and environmental issues in show caves. World J Microbiol Biotechnol. 2012; 28(7): 2453–2464. doi: 10.1007/s11274-012-1070-x 22806150 10.1007/s11274-012-1070-x

[14] Schabereiter-Gurtner C, Piñar G, Lubitz W, Rölleke S. An advanced molecular strategy to identify bacterial communities on art objects. J Microbiol Methods. 2001; 45(2):77–87. 11311392 10.1016/s0167-7012(01)00227-5

[15] Otlewska A, Adamiak J, Gutarowska B. Application of molecular techniques for the assessment of microorganism diversity on cultural heritage objects. Acta Biochim Pol.2014; 61(2): 217–225. 24927237

[16] Santos A, Cerrada A, García S, San Andrés M, Abrusci C, Marquina D. Application of molecular techniques to the elucidation of the microbial community structure of antique paintings. Microb Ecol. 2009; 58(4): 692–702. doi: 10.1007/s00248-009-9564-2 19633806 10.1007/s00248-009-9564-2

[17] Konkol NR, Mcnamara CJ, Hellman E, Mitchell R. Early detection of fungal biomass on library materials. J Cult Herit. 2012; 13(2): 115–119.

[18] Vivar I, Borrego S, Ellis G, Moreno DA, García AM. Fungal biodeterioration of color cinematographic films of the cultural heritage of Cuba. Int Biodeter Biodegr. 2013; 84(5): 372–380.

[19] Sterflinger K. Fungi: Their role in deterioration of cultural heritage. Fungal Biol Rev. 2010; 24(1–2): 47–55.

[20] Gu J-D. Microbiological deterioration and degradation of synthetic polymeric materials: recent research advances. Int Biodeter Biodegr. 2003; 52(2): 69–91.

[21] Sterflinger K, Piñar G. Microbial deterioration of cultural heritage and works of art—tilting at windmills? Appl Microbiol Biotechnol.2013; 97(22): 9637–9646. doi: 10.1007/s00253-013-5283-1 24100684 10.1007/s00253-013-5283-1 PMC3825568

[22] Feng QP, Ma XJ, Zhang X, Li Z, Li S. Studies on microbial factor on color change of Dunhuang mural. I. Classification of microbes on color-changed mural and property of some typical species. Acta Microbiol Sin. 1998; 38(2): 52–56. 12549389

[23] Feng QP, Zhang X, Ma Q, Ma XJ. Studies on microbiological factor in colour change of Mogao Grottoes' mural. II. Effect of microorganism on the pigment of imitative mural. Acta Microbiol Sin. 1998; 38(2):131–136. 12549374

[24] Wang WF, Ma YT, Ma X, Wu FS, Ma XJ, An LZ, et al Seasonal variations of airborne bacteria in the Mogao Grottoes, Dunhuang, China. Int Biodeter Biodegr. 2010; 64(4): 309–315.

[25] Wang WF, Ma X, Ma YT, Mao L, Wu FS, Ma XJ, et al Seasonal dynamics of airborne fungi in different caves of the Mogao Grottoes, Dunhuang, China. Int Biodeter Biodegr. 2010; 64(6): 461–466.

[26] Wang WF, Ma X, Ma YT, Mao L, Wu FS, Ma XJ, et al Molecular characterization of airborne fungi in caves of the Mogao Grottoes, Dunhuang, China. Int Biodeter Biodegr. 2011; 65(5): 726–731.

[27] Wang WF, Ma YT, Ma X, Wu F, Ma XJ, An LZ, et al Diversity and seasonal dynamics of airborne bacteria in the Mogao Grottoes, Dunhuang, China. Aerobiologia. 2012; 28(1): 27–38.

[28] Bastian F, Jurado V, Nováková A, Alabouvette C, Saiz-jimenez C. The microbiology of Lascaux Cave. Microbiology. 2010; 156(Pt3): 644–652. 20056706 10.1099/mic.0.036160-0

[29] Dennis KL, Wang Y, Blatner NR, Wang S, Saadalla A, Trudeau E, et al Adenomatous polyps are driven by microbe-instigated focal inflammation and are controlled by IL-10 producing T-cells. Cancer Res. 2013; 73(19): 5905–5913. doi: 10.1158/0008-5472.CAN-13-1511 23955389 10.1158/0008-5472.CAN-13-1511 PMC4322779

[30] Rousk J, Bååth E, Brookes PC, Lauber CL, Lozupone C, Caporaso JG, et al Soil bacterial and fungal communities across a pH gradient in an arable soil. ISME J. 2010; 4(10): 1340–1351. doi: 10.1038/ismej.2010.58 20445636 10.1038/ismej.2010.58

[31] Quast C, Pruesse E, Yilmaz P, Gerken J, Schweer T, Yarza P, et al The SILVA ribosomal RNA gene database project: improved data processing and web-based tools. Nucleic Acids Res. 2013; 41: 590–596. 10.1093/nar/gks1219 PMC3531112 23193283

[32] Schloss PD, Westcott SL, Ryabin T, Hall JR, Hartmann M, Hollister EB, et al Introducing mothur: open-source, platform-independent, community-supported software for describing and comparing microbial communities. Appl Environ Microbiol. 2009; 75(23): 7537–7541. doi: 10.1128/AEM.01541-09 19801464 10.1128/AEM.01541-09 PMC2786419

[33] Jurado V, Groth I, Gonzalez JM, Laiz L, Saiz-jimenez C. Agromyces salentinus sp. nov. and Agromyces neolithicus sp. nov. Int J Syst Evol Microbiol. 2005; 55(Pt1): 153–157. 15653869 10.1099/ijs.0.63199-0

[34] Jurado V, Groth I, Gonzalez JM, Laiz L, Saiz-Jimenez C. Agromyces subbeticus sp. nov., isolated from a cave in southern Spain. Int J Syst Evol Microbiol. 2005; 55(Pt5): 1897–1901. 16166685 10.1099/ijs.0.63637-0

[35] Jurado V, Boiron P, Kroppenstedt RM, Laurent F, Couble A, Laiz L, Klenk HP, et al *Nocardia altamirensis* sp. nov., isolated from Altamira cave, Cantabria, Spain. Int J Syst Evol Microbiol. 2008; 58(9): 2210–2214. 18768631 10.1099/ijs.0.65482-0

[36] Lee NM, Meisinger DB, Aubrecht R, Kovacik L, Saiz-jimenez C, Baskar R, et al Caves and karst environments Life at Extremes: Environments, Organisms and Strategies for Survival. CAB International, UK 2012; p.320–344.

[37] Laiz L, Groth I, Gonzalez I, Saiz-Jimenez C. Microbiological study of the dripping waters in Altamira cave (Santillana del Mar, Spain). J Microbiol Methods. 1999; 36(1–2):129–138. 10353807 10.1016/s0167-7012(99)00018-4

[38] Stomeo F, Portillo MC, Gonzalez JM, Laiz L, Saiz-Jimenez C. *Pseudonocardia* in white colonizations in two caves with Paleolithic paintings. Int Biodeter Biodegr. 2008; 62(4):483–486.

[39] Wu FS, Wang WF, He DP, Chen GQ, Ma YT, Zhang XD, et al Molecular techniques used to analyze the bacterial groups on mural paintings in Wei and Jin Dynasty Tombs, Jiayuguan. Dunhuang Res.2011; 6: 51–58.

[40] Portillo MC, Gonzalez JM, Saiz-Jimenez C. Metabolically active microbial communities of yellow and grey colonizations on the walls of Altamira Cave, Spain. J Appl Microbiol. 2008; 104(3): 681–691. doi: 10.1111/j.1365-2672.2007.03594.x 17927740 10.1111/j.1365-2672.2007.03594.x

[41] Imperi F, Caneva G, Cancellier L, Ricci MA, Sodo A, Visca P. The bacterial aetiology of rosy discoloration of ancient wall paintings. Environ Microbiol. 2007; 9(11): 2894–2902. doi: 10.1111/j.1462-2920.2007.01393.x 17922771 10.1111/j.1462-2920.2007.01393.x

[42] Nugari MP, Pietrini AM, Caneva G, Imperi F, Visca P. Biodeterioration of mural paintings in a rocky habitat: The Crypt of the Original Sin (Matera, Italy). Int Biodeter Biodegr. 2009; 63(6): 705–711.

[43] Laiz L, Miller AZ, Jurado V, Akatova E, Sanchez-Moral S, Gonzalez JM, et al Isolation of five *Rubrobacter* strains from biodeteriorated monuments. Sci Nat. 2009; 96(1): 71–79. 10.1007/s00114-008-0452-2 18953520

[44] Kusumi A, Li X, Osuga Y, Kawashima A, Gu J-D, Nasu M, et al Bacterial communities in pigmented biofilms formed on the sandstone Bas-Relief walls of the Bayon Temple, Angkor Thom, Cambodia. Microbes Environ. 2013; 28(4): 422–431. doi: 10.1264/jsme2.ME13033 24334526 10.1264/jsme2.ME13033 PMC4070708

[45] Schabereiter-Gurtner C, Piñar G, Vybiral D, Lubitz W, Rölleke S. *Rubrobacter*-related bacteria associated with rosy discolouration of masonry and lime wall paintings. Arch Microbiol. 2001; 176(5):347–354. doi: 10.1007/s002030100333 11702076 10.1007/s002030100333

[46] Bassi M, Ferrari A, Realini M, Sorlini C. Red stains on the Certosa of Pavia: A case of biodeterioration. Int Biodeter Biodegr. 1986; 22(3):201–205.

[47] Ciferri O. Microbial degradation of paintings. Appl Environ Microbiol. 1999; 65(3):879–885. 10049836 10.1128/aem.65.3.879-885.1999 PMC91117

[48] Heyrman J, Mergaert J, Denys R, Swings J. The use of fatty acid methyl ester analysis (FAME) for the identification of heterotrophic bacteria present on three mural paintings showing severe damage by microorganisms. FEMS Microbiol Lett. 1999; 181(1):55–62. 10564789 10.1111/j.1574-6968.1999.tb08826.x

[49] Pangallo D, Kraková L, Chovanová K, Simonovičová A, De Leo F, Urzì C. Analysis and comparison of the microflora isolated from fresco surface and from surrounding air environment through molecular and biodegradative assays. World J Microbiol Biotechnol. 2012; 28(5):2015–2027. doi: 10.1007/s11274-012-1004-7 22806023 10.1007/s11274-012-1004-7

[50] De Leo F, Iero A, Zammit G, Urzi Clara E. Chemoorganotrophic bacteria isolated from biodeteriorated surfaces in cave and catacombs. Int J Speleol. 2012; 41(41):1–12.

[51] Bastian F, Alabouvette C, Jurado V, Saiz-Jimenez C. Impact of biocide treatments on the bacterial communities of the Lascaux Cave. Sci Nat. 2009; 96(7):863–868. 10.1007/s00114-009-0540-y 19404600

[52] Yan F, Ge QY, Li Q, Yu M, Zhu XD, Pan J, et al Analysis of microbial community on the surface of the historic stone and nearby rock samples in Yungang Grottoes. Acta Microbiol Sin. 2012; 52(5):629–636. 22803349

[53] Ettenauer J, Piñar G, Sterflinger K, Gonzalez-Muñoz MT, Jroundi F. Molecular monitoring of the microbial dynamics occurring on historical limestone buildings during and after the in situ application of different bio-consolidation treatments. Sci Total Environ. 2011; 409(24): 5337–5352. doi: 10.1016/j.scitotenv.2011.08.063 21944202 10.1016/j.scitotenv.2011.08.063 PMC3209562

[54] Jroundi F, Fernández-Vivas A, Rodriguez-Navarro C, Bedmar EJ, González-Muñoz MT. Bioconservation of deteriorated monumental calcarenite stone and identification of bacteria with carbonatogenic activity. Microb Ecol. 2010; 60(1):39–54. doi: 10.1007/s00248-010-9665-y 20386895 10.1007/s00248-010-9665-y

[55] Piñar G, Jimenez-Lopez C, Sterflinger K, Ettenauer J, Jroundi F, Fernandez-Vivas A, et al Bacterial community dynamics during the application of a *Myxococcus xanthus*-inoculated culture medium used for consolidation of ornamental limestone. Microb Ecol. 2010; 60(1):15–28. doi: 10.1007/s00248-010-9661-2 20393845 10.1007/s00248-010-9661-2 PMC2917555

[56] Fouhy F, Clooney AG, Stanton C, Claesson MJ, Cotter PD. 16S rRNA gene sequencing of mock microbial populations-impact of DNA extraction method, primer choice and sequencing platform. BMC Microbiol. 2016; 16(1): 123 doi: 10.1186/s12866-016-0738-z 27342980 10.1186/s12866-016-0738-z PMC4921037

[57] Mueller RC, Gallegos-Graves LV, Kuske CR. A new fungal large subunit ribosomal RNA primer for high throughput sequencing surveys. FEMS Microbiol Ecol; 2016; 92(2): fiv153 doi: 10.1093/femsec/fiv153 26656064 10.1093/femsec/fiv153

[58] Saiz-jimenez C, Cuezva S, Jurado V, Fernandez-Cortes A, Porca E, Benavente D, et al Conservation. Paleolithic art in peril: policy and science collide at Altamira Cave. Science. 2011; 334(6052):42–43. doi: 10.1126/science.1206788 21980097 10.1126/science.1206788

[59] Sánchez-Moral S, Soler V, Cañaveras JC, Sanz-Rubio E, Van GR, Gysels K. Inorganic deterioration affecting the Altamira Cave, N Spain: quantitative approach to wall-corrosion (solutional etching) processes induced by visitors. Sci Total Environ. 1999; 243–244:67–84. 10635591 10.1016/s0048-9697(99)00348-4

[60] Jurado V, Laiz L, Rodriguez-Nava V, Boiron P. Pathogenic and opportunistic microorganisms in caves. Int J Speleol. 2010; 39(1):15–24.

[61] Viles HA, Cutler NA. Global environmental change and the biology of heritage structures. Global Change Biol. 2012; 18(8):2406–2418.

