# Correction: The microbial community characteristics of ancient painted sculptures in Maijishan Grottoes, China

**DOI:** 10.1371/journal.pone.0183598

**Published:** 2017-08-15

**Authors:** Yulong Duan, Fasi Wu, Wanfu Wang, Dongpeng He, Ji-Dong Gu, Huyuan Feng, Tuo Chen, Guangxiu Liu, Lizhe An

The second affiliation for the first author is missing. Yulong Duan is affiliated with #1 Key Laboratory of Extreme Environmental Microbial Resources and Engineering, Gansu Province, Northwest Institute of Eco-Environment and Resources, University of Chinese Academy of Sciences, Lanzhou, P.R.China and #5 University of Chinese Academy of Sciences, Beijing, 100049, P.R.China.
