# Correction: Correction: The microbial community characteristics of ancient painted sculptures in Maijishan Grottoes, China

**DOI:** 10.1371/journal.pone.0194902

**Published:** 2018-03-22

**Authors:** The PLOS ONE Staff

There is an error in the Correction published on August 15, 2017. There is an error in affiliation 1. The publisher apologizes for the error. The correct text is:

The second affiliation for the first author is missing. Yulong Duan is affiliated with #1 Key Laboratory of Extreme Environmental Microbial Resources and Engineering, Gansu Province, Northwest Institute of Eco-Environment and Resources, Chinese Academy of Sciences, Lanzhou, P.R.China and #5 University of Chinese Academy of Sciences, Beijing, P.R.China.
