# Supplementary figures and images for: The microbial community characteristics of ancient painted sculptures in Maijishan Grottoes, China
Source: PLoS One. 2017 Jul 5;12(7):e0179718. doi: 10.1371/journal.pone.0179718 (PMC5497971; doi:10.1371/journal.pone.0179718)

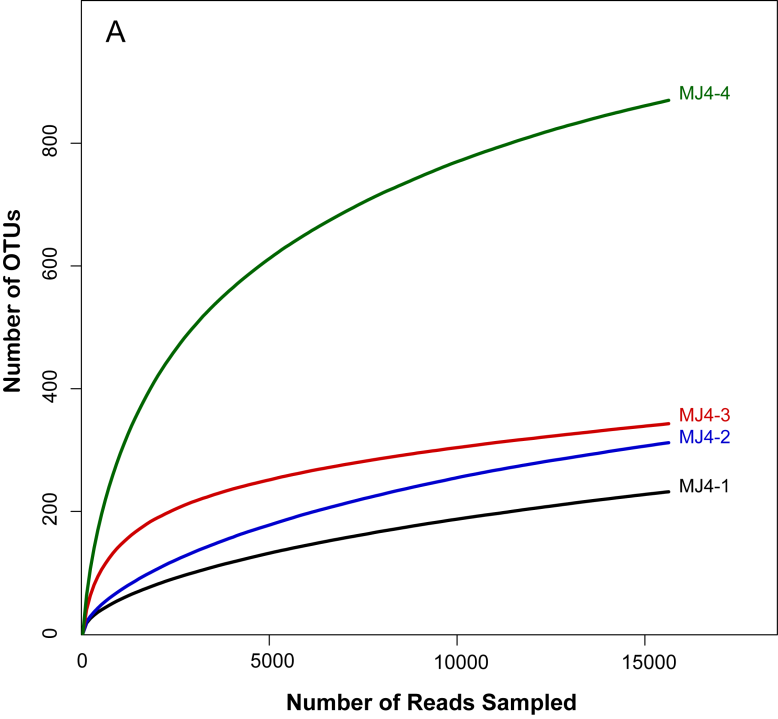

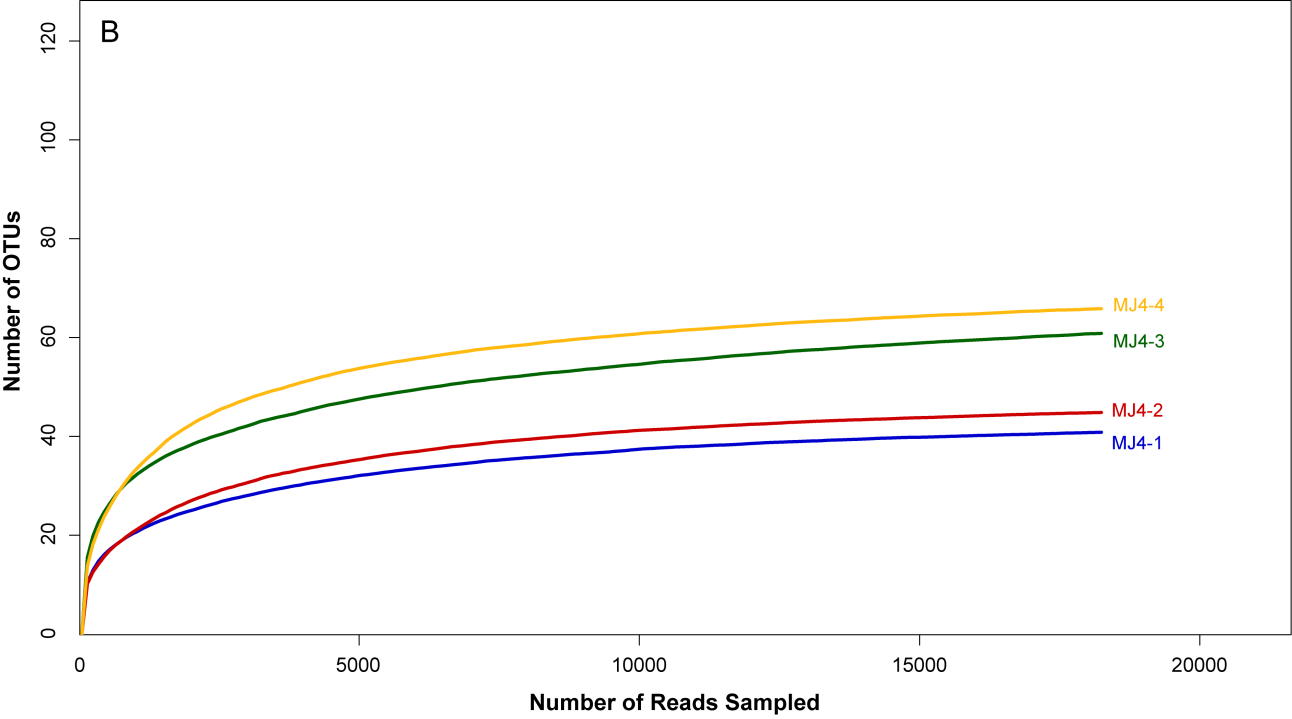

Supplement: S1 Fig — Rarefaction curves show the number of reads with the number of phylotypes at 97% sequence similarity level for the different samples. A: Bacteria, B: Fungi. (DOCX) [file pone.0179718.s001.docx]
