# Supplementary material for: The microbial community characteristics of ancient painted sculptures in Maijishan Grottoes, China
Source: PLoS One. 2017 Jul 5;12(7):e0179718. doi: 10.1371/journal.pone.0179718 (PMC5497971; doi:10.1371/journal.pone.0179718)
Supplement: S1 Table — The operational taxonomic units (OTUs) were defined with 97% similarity threshold. The coverage percentages, richness estimators (ACE and Chao), and diversity indices (Shannon and Simpson) were calculated. (DOCX) [file pone.0179718.s002.docx]

|  | | | | | | | | | |
| --- | --- | --- | --- | --- | --- | --- | --- | --- | --- |
| Samples | **Valid sequence** | **Average length (bp)** | **Reads** | **OTUs** | **Ace** | **Chao** | **Shannon** | **Simpson** | **Coverage (%)** |
| MJ4-1 | 36,378 | 437.21 | 15,595 | 232 | 546 | 367 | 2.15 | 0.2665 | 99.31 |
| MJ4-2 | 37,299 | 440.89 | 15,595 | 312 | 481 | 456 | 2.57 | 0.1543 | 99.15 |
| MJ4-3 | 38,038 | 435.30 | 15,595 | 343 | 500 | 489 | 3.42 | 0.1563 | 99.39 |
| MJ4-4 | 21,369 | 434.25 | 15,595 | 870 | 1,059 | 1,039 | 5.12 | 0.0208 | 98.60 |
