# Supplementary material for: The microbial community characteristics of ancient painted sculptures in Maijishan Grottoes, China
Source: PLoS One. 2017 Jul 5;12(7):e0179718. doi: 10.1371/journal.pone.0179718 (PMC5497971; doi:10.1371/journal.pone.0179718)
Supplement: S2 Table — The operational taxonomic units (OTUs) were defined with 97% similarity threshold. The coverage percentages, richness estimators (ACE and Chao), and diversity indices (Shannon and Simpson) were calculated. (DOCX) [file pone.0179718.s003.docx]

|  | | | | | | | | | |
| --- | --- | --- | --- | --- | --- | --- | --- | --- | --- |
| Samples | **Valid sequence** | **Average length (bp)** | **Reads** | **OTUs** | **Ace** | **Chao** | **Shannon** | **Simpson** | **Coverage (%)** |
| MJ4-1 | 22,813 | 402.87 | 18,193 | 42 | 45 | 44 | 2.1 | 0.1549 | 99.97 |
| MJ4-2 | 26,782 | 402.94 | 18,193 | 46 | 49 | 49 | 1.72 | 0.3152 | 99.97 |
| MJ4-3 | 31,041 | 402.87 | 18,193 | 62 | 71 | 70 | 2.41 | 0.1334 | 99.94 |
| MJ4-4 | 20,379 | 402.91 | 18,193 | 67 | 72 | 73 | 1.88 | 0.3176 | 99.95 |
