# Supplementary material for: The microbial community characteristics of ancient painted sculptures in Maijishan Grottoes, China
Source: PLoS One. 2017 Jul 5;12(7):e0179718. doi: 10.1371/journal.pone.0179718 (PMC5497971; doi:10.1371/journal.pone.0179718)
Supplement: S3 Table — (DOCX) [file pone.0179718.s004.docx]

|  | | | | | | | | | |
| --- | --- | --- | --- | --- | --- | --- | --- | --- | --- |
| Phylum | **Shared OTUs** | **Reads of shared OTUs** | | | | **Reads of shared OTUs/Total reads (%)** | | | |
|  |  | **MJ4-1** | **MJ4-2** | **MJ4-3** | **MJ4-4** | **MJ4-1** | **MJ4-2** | **MJ4-3** | **MJ4-4** |
| *Actinobacteria* | **31** | **14,258** | **12,924** | **9,133** | **3,856** | **91.42** | **82.87** | **58.56** | **24.72** |
| *Bacteroidetes* | 2 | 2 | 6 | 2 | 35 | 0.013 | 0.038 | 0.013 | 0.22 |
| *Cyanobacteria* | 2 | 30 | 60 | 18 | 1,846 | 0.19 | 0.39 | 0.12 | 11.84 |
| *Firmicutes* | **27** | **684** | **1,413** | **667** | **672** | **4.38** | **9.06** | **4.28** | **4.31** |
| *Proteobacteria* | 16 | 160 | 469 | 33 | 1,251 | 1,02 | 3.10 | 0.21 | 8.02 |
| *Total shared sequences* | 78 | 15,134 | 14,872 | 9,853 | 7,660 | 97.04 | 95.36 | 63.18 | 49.12 |
