# Supplementary material for: The microbial community characteristics of ancient painted sculptures in Maijishan Grottoes, China
Source: PLoS One. 2017 Jul 5;12(7):e0179718. doi: 10.1371/journal.pone.0179718 (PMC5497971; doi:10.1371/journal.pone.0179718)
Supplement: S4 Table — (DOCX) [file pone.0179718.s005.docx]

|  | | | | | | | | | |
| --- | --- | --- | --- | --- | --- | --- | --- | --- | --- |
| Phylum | **Shared OTUs** | **Reads of shared OTUs** | | | | **Reads of shared OTUs/Total reads (%)** | | | |
|  |  | **MJ4-1** | **MJ4-2** | **MJ4-3** | **MJ4-4** | **MJ4-1** | **MJ4-2** | **MJ4-3** | **MJ4-4** |
| *Ascomycota* | **26** | **17,915** | **16,889** | **16,709** | **17,195** | **94.51** | **92.83** | **91.84** | **94.51** |
| *Basidiomycota* | 8 | 253 | 1,275 | 1,327 | 775 | 1.39 | 7.01 | 7.29 | 4.26 |
| *Total shared sequences* | 34 | 18,168 | 18,164 | 18,036 | 17,970 | 99.86 | 99.84 | 99.14 | 98.77 |
