# Supplementary material for: The microbial community characteristics of ancient painted sculptures in Maijishan Grottoes, China
Source: PLoS One. 2017 Jul 5;12(7):e0179718. doi: 10.1371/journal.pone.0179718 (PMC5497971; doi:10.1371/journal.pone.0179718)
Supplement: S5 Table — Eighteen OTUs with abundances higher than 0.5% in the bacterial community were sorted from total of 1,757 OTUs, and defined as predominant OTUs. S: = species; G: = genus; F: = family; O: = order; C: = class; P: = phylum. (DOCX) [file pone.0179718.s006.docx]

|  | | | | | |
| --- | --- | --- | --- | --- | --- |
| OTUs | **Samples** | | | | **Annotation ^2^** |
|  | **MJ4-1** | **MJ4-2** | **MJ4-3** | **MJ4-4** |  |
| OTU352 | 7,462 | 2,953 | 5,970 | 573 | G: *Pseudonocardia* |
| OTU1409 | 2,541 | 4,607 | 409 | 228 | G: *Pseudonocardia* |
| OTU989 | 1,007 | 2,249 | 874 | 185 | G: *Rubrobacter* |
| OTU926 | 324 | 269 | 600 | 1,387 | G: *Saccharopolyspora* |
| OTU1657 | 884 | 897 | 549 | 219 | G: *Actinomycetospora* |
| OTU62 | 583 | 552 | 378 | 160 | G: *Actinomycetospora* |
| OTU1040 | 195 | 708 | 10 | 121 | G: G*eobacillus* |
| OTU1009 | 1 | 25 | 10 | 947 | C: *Cyanobacteria* |
| OTU1322 | 29 | 35 | 8 | 899 | C: *Cyanobacteria* |
| OTU1828 | 162 | 696 | 9 | 12 | G: *Crossiella* |
| OTU1290 | 157 | 445 | 9 | 119 | G: *Lactococcus* |
| OTU101 | 420 | 20 | 7 | 16 | G: *Brevibacterium* |
| OTU1218 | 97 | 269 | 6 | 79 | G: *Pseudomonas* |
| OTU545 | 0 | 3 | 2 | 378 | S: *Pseudomonas* |
| OTU592 | 199 | 25 | 107 | 42 | F: *Streptomycetaceae* |
| OTU218 | 0 | 10 | 7 | 330 | C: *Cyanobacteria* |
| OTU1099 | 1 | 0 | 271 | 51 | O: *Bacteroidales* |
| OTU962 | 116 | 85 | 66 | 45 | G: *Pseudonocardia* |
