# Supplementary material for: The microbial community characteristics of ancient painted sculptures in Maijishan Grottoes, China
Source: PLoS One. 2017 Jul 5;12(7):e0179718. doi: 10.1371/journal.pone.0179718 (PMC5497971; doi:10.1371/journal.pone.0179718)
Supplement: S6 Table — Fifteen OTUs with abundances higher than 0.5% in the fungal community were sorted from total of 217 OTUs, and defined as predominant OTUs. S: = species; G: = genus; F: = family; O: = order; C: = class; P: = phylum. (DOCX) [file pone.0179718.s007.docx]

|  | | | | | |
| --- | --- | --- | --- | --- | --- |
| OTUs | **Samples** | | | | **Annotation** |
|  | **MJ4-1** | **MJ4-2** | **MJ4-3** | **MJ4-4** |  |
| OTU200 | 4,195 | 9,650 | 2,204 | 9,815 | O: *Capnodiales* |
| OTU10 | 3,376 | 2,025 | 3,684 | 2,370 | O: *Capnodiales* |
| OTU117 | 3,415 | 1,966 | 1,804 | 426 | P: *Ascomycota* |
| OTU155 | 1,160 | 1,097 | 4,183 | 981 | O: *Capnodiales* |
| OTU182 | 1,978 | 841 | 1,714 | 505 | O: *Eurotiales* |
| OTU147 | 2,186 | 403 | 912 | 76 | F: *Trichocomaceae* |
| OTU220 | 170 | 953 | 282 | 593 | O: *Exobasidiales* |
| OTU170 | 397 | 389 | 460 | 542 | P: *Ascomycota* |
| OTU29 | 130 | 161 | 640 | 226 | P: *Ascomycota* |
| OTU99 | 40 | 264 | 518 | 18 | P: *Basidiomycota* |
| OTU219 | 6 | 51 | 40 | 717 | S: *Bipolaris sorokiniana* |
| OTU142 | 15 | 33 | 91 | 605 | S: *Torula herbarum* |
| OTU148 | 595 | 16 | 73 | 7 | F: *Microascaceae* |
| OTU71 | 7 | 29 | 151 | 396 | C: *Dothideomycetes* |
| OTU7 | 113 | 24 | 237 | 109 | F: *Herpotrichiellaceae* |
